# Role of *HLA-DP* Polymorphisms on Chronicity and Disease Activity of Hepatitis B Infection in Southern Chinese

**DOI:** 10.1371/journal.pone.0066920

**Published:** 2013-06-25

**Authors:** Danny Ka-Ho Wong, Tsunamasa Watanabe, Yasuhito Tanaka, Wai-Kay Seto, Cheuk-Kwong Lee, James Fung, Che-Kit Lin, Fung-Yu Huang, Ching-Lung Lai, Man-Fung Yuen

**Affiliations:** 1 Department of Medicine, The University of Hong Kong, Queen Mary Hospital, Hong Kong; 2 State Key Laboratory for Liver Research, The University of Hong Kong, Queen Mary Hospital, Hong Kong; 3 Department of Virology and Liver Unit, Nagoya City University Graduate School of Medical Sciences, Nagoya, Japan; 4 Hong Kong Red Cross Blood Transfusion Service, Hospital Authority, Hong Kong; University of Modena & Reggio Emilia, Italy

## Abstract

**Background and Aims:**

The association between *HLA-DP* single nucleotide polymorphisms (SNPs) and chronic hepatitis B virus (HBV) infection varies between different populations. We aimed to study the association between *HLA-DP* SNPs and HBV infection and disease activity in the Chinese population of Hong Kong.

**Methods:**

We genotyped SNPs rs3077 (near *HLA-DPA1*) and rs9277378 and rs3128917 (both near *HLA-DPB1*) in 500 HBV carriers (hepatitis B surface antigen [HBsAg]-positive), 245 non-HBV infected controls (HBsAg- and antibody to hepatitis B core protein [anti-HBc]-negative), and 259 subjects with natural HBV clearance (HBsAg-negative, anti-HBc-positive). Inactive HBV carriers state was defined by HBV DNA levels <2,000 IU/ml and persistently normal alanine aminotransferase level for least 12 months.

**Results:**

Compared to the non-HBV infected subjects, the HBV carriers had a significantly lower frequency of the rs3077 T allele (p = 0.0040), rs9277378 A allele (p = 0.0068) and a trend for lower frequency of rs3128917 T allele (p = 0.054). These alleles were associated with an increased chance of HBV clearance (rs3077: OR = 1.41, p = 0.0083; rs9277378: OR = 1.61, p = 0.00011; rs3128917: OR = 1.54, p = 0.00017). Significant associations between HLA-DP genotypes and HBV clearance were also found under different genetic models. Haplotype TAT was associated with an increased chance of HBV clearance (OR = 1.64, p = 0.0013). No association was found between these SNPs and HBV disease activity.

**Conclusion:**

*HLA-DP* SNPs rs3077, rs9277378 and rs3128917 were associated with chronicity of HBV disease in the Chinese. Further studies are required to determine whether these SNPs influence the disease endemicity in different ethnic populations.

## Introduction

Approximately 400 million people worldwide are chronic carriers of hepatitis B virus (HBV) [1]. The disease spectrum of chronic hepatitis B varies among patients, ranging from inactive non-replicative to active replicative state, which may lead to fulminant hepatic failure, liver cirrhosis, or hepatocellular carcinoma (HCC). While persistence or resolution of HBV infection may be affected by a variety of factors, including viral, environmental and host factors, family or twin studies have suggested that host genetic constitution is also an important factor which influences chronicity of HBV infection [2], [3]. Many host genetic variations, including genes coding for cytokines such as interferon-gamma and tumor necrosis factors [4], estrogen receptor alpha [5], vitamin D receptor [6], mannose-binding protein [7], cytotoxic T-lymphocyte antigen 4 (*CTLA-4*) [8] and human leukocyte antigen (HLA) [9], [10], [11], [12], have been suggested to influence chronicity or clearance of HBV infection. In particular, single nucleotide polymorphisms (SNPs) near the *CTLA-4*, genes coding for an inhibitory receptor expressed by T-lymphocytes, and near the *HLA-DR13* locus, coding for component of the major histocompatibility complex class II cell surface receptors, have been studied in several case-control studies for their association with HBV infection in different populations [8], [9], [10], [11], [12]. However, these candidate gene studies were not conducted on a large scale genome-wide approach.

Several genome-wide association studies (GWAS) have been performed with large cohorts to study the association of genetic variations with HBV infection. These GWAS studies did not find a strong association between HBV infection and those previously identified candidate HBV-associated SNPs. These GWAS studies demonstrated that certain SNPs near the *HLA-DP* loci, are associated with persistent HBV infection [13], [14], [15]. In a pioneering GWAS study with 786 Japanese chronic HBV carriers and 2,201 controls, Kamatani and colleagues have identified an association between chronic hepatitis B and 11 SNPs in the *HLA-DP* region, two of which, namely rs3077 and rs9277535, were further validated in three additional Japanese and Thai cohorts [13]. The association between these *HLA-DP* SNPs with chronicity and/or clearance of HBV infection was further confirmed by two other GWAS studies, one with 2,667 Japanese chronic HBV carriers and 6,496 controls by the same group [14] and one with 181 Japanese chronic HBV carries, 184 healthy controls, and 185 individuals with natural clearance of HBV [15]. The association of some of these *HLA-DP* SNPs with HBV infection has been verified in many studies, but the association differs between studies in different population cohorts, and more SNPs are yet to be identified [16], [17], [18], [19], [20], [21].

HLA-DP molecules, belonging to HLA class II, are involved in antigen presentation to CD4+ T helper cells. As HLA-DP plays an important role in host-immune response and particularly antigen presentation, it would be interesting to investigate the possible association of the *HLA-DP* loci variations with hepatitis B disease activity, which is immune-mediated. Since the findings are not consistent in different study cohorts [18], [20], the association of these *HLA-DP* SNPs with HBV disease activity remains unclear.

In the present study, we primarily aimed to investigate the association of 3 *HLA-DP* SNPs, namely rs3077, rs9277378 and rs3128917, with chronicity of HBV infection in the Chinese population in Hong Kong. In addition, we studied the association of these SNPs with hepatitis B disease activity. This will extend our understanding of the association between *HLA-DP* variations and HBV infection and may provide some evidences to explain the widely different prevalence of chronic HBV in different ethnic groups in the world.

## Patients and Methods

### Patients

The present study recruited 500 chronic HBV carriers (hepatitis B surface antigen [HBsAg]-positive for more than 6 months) who had been followed up in our liver clinics in the Queen Mary Hospital, Hong Kong. Upon their first and/or follow up visits, these HBV carriers had given verbal informed consent for the storage of blood samples for further studies. We have also recruited 706 consecutive HBsAg-negative control subjects who have donated blood at the Hong Kong Red Cross Blood Transfusion Service, and they all had given verbal informed consent during blood donation for the storage of blood samples. Data were analyzed anonymously for the 706 HBsAg-negative blood donors. Approval has been obtained from the Institution Review Board, Queen Mary Hospital, The University of Hong Kong, for retrieving archived samples for this study. Among the 706 HBsAg-negative subjects, 202 had previous history of hepatitis B vaccination and were excluded from the subsequent analysis. All study patients/subjects are Chinese, and all blood samples were collected during the period January 2010 to March 2011. All subjects were tested negative for hepatitis C virus and human immunodeficiency virus by the Procleix Ultrio Assay (Novartis Diagnostics, Emeryville, CA).

Of the 504 non-vaccinated control subjects, 259 had HBV natural clearance (HBV clearance group), denoted by the presence of detectable anti-HBc by the Elecsys assay (Roche Diagnostics, Basel, Switzerland). The remaining 245 subjects (non-HBV infected group) were negative for both HBsAg and anti-HBc. Longitudinal clinical data, including alanine aminotransferase (ALT) and HBV DNA measurements, were obtained from the 500 HBV carriers. Inactive asymptomatic HBV carriers were defined by HBV DNA levels <2,000 IU/ml and persistently normal ALT (<58 U/L for male and <36 U/L for female) for least 12 months.

### Genotyping Assays

**Figure 1:**
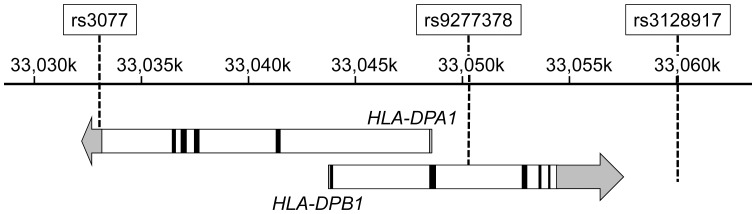
Relative locations of the three studied SNPs and *HLA-DPA1* and *HLA-DPB1* genes on chromosome 6. The names of the three SNPs are shown on top, and the chromosomal positions on chromosome 6 are marked in the ruler in the middle. The *HLA-DPA1* and *HLA-DPB1* genes are shown as arrows in the bottom, with the exons shown as black boxes, introns as white boxes, and un-translated regions as gray boxes/arrows.

Three SNPs within chromosome 6, namely rs3077 (in the 3′ untranslated region of the *HLA-DPA1* gene), rs9277378 and rs3128917 (inside and near the *HLA-DPB1* gene, respectively), were studied (Figure 1). rs3077 was selected for this study because rs3077 and rs9277535 (at the 3′ untranslated region of *HLA-DPB1*) have been identified to be strongly associated with persistent HBV infection [13]. We chose to study rs9277378 instead of rs9277535 because, our previous large scale genotypic analysis revealed that, rs9277378 was more readily detected in DNA extracted from sera than rs9277535 (data not shown). Moreover, linkage disequilibrium (LD) analysis by the Haploview software (version 4.2) revealed that rs9277378 has a strong LD with rs9277535 (D’ = 1.00, R^2^ = 0.954) in the HapMap Han Chinese in Beijing (CHB) and Japanese in Tokyo (JPT) Populations. [22] We also confirmed, in a small subset of 50 randomly selected samples from the current study, that rs9277535 and rs9277378 genotypes were concordant in 48 (96%) samples with a strong LD (D’ = 1.00, R^2^ = 0.903). The SNP rs3128917 was also chosen for the present study, as this SNP has the highest odds ratio (OR) among 11 SNPs which influence chronicity of HBV infection [16].

The 3 selected SNPs, rs3077, rs9277378 and rs3128917, were genotyped using the TaqMan SNP genotyping assay (Life Technologies, Carlsbad, CA). Briefly, free circulating DNA was extracted from 200 µl of serum samples, using the Purelink Genomic DNA Mini Kit (Life Technologies). The SNP genotyping reaction was performed in a TaqMan real-time PCR format, using SNP-specific primers and FAM- and VIC-labeled allele-specific probes provided in the TaqMan SNP genotyping kit (Life Technologies) and the real-time PCR enzymes and reagents provided in QuantiFast Probe PCR Kit (QIAGEN, Hilden, Germany). The real-time PCR reaction was performed in a RotorGene-Q real-time PCR System (QIAGEN).

### Statistical Analyses

Statistical analyses were performed using PLINK v.1.07 (http://pngu.mgh.harvard.edu/purcell/plink/) [23] and SPSS 18.0 (SPSS Inc., Chicago, IL). Logistic regression was performed to compare between case and control groups, and all ORs and p values were adjusted for age and sex. The Student *t* test was used to test normally distributed variables. Categorical variables were tested by the Chi-square test. Statistical significance was defined by p<0.05.

## Results

### Demographic Characteristics and Hardy-Weinberg Equilibrium

The mean age of the HBV carriers was 46.8±12.1 years, which was significantly higher than that of the non-HBV infected subjects (36.4±9.9 years; p<0.0001) and that of the HBV clearance subjects (40.3±10.9 years; p<0.0001). The proportion of male (304/500; 61%) in the HBV carriers was significantly higher than that in the non-HBV infected group (127/245; 52%; p = 0.020), but did not differ from that in the HBV clearance subjects (152/259; 59%; p = 0.573). Three SNPs (rs3077, rs9277378, and rs3128917) were genotyped in these 500 HBV carriers, 245 non-HBV infected controls and 259 HBV clearance subjects. All 3 polymorphisms in the HBV carriers, non-HBV infected controls and HBV clearance subjects were in Hardy-Weinberg equilibrium, and there was no significant difference between the observed and expected genotypic frequencies in all 3 SNPs in all 3 groups (all p>0.05; Table S1).

### Association between HLA-DP Polymorphisms and Chronicity of HBV Infection

The allelic frequencies of the three studied SNPs are listed in Table 1. The minor alleles for rs3077, rs9277378 and rs3128917 determined from the present study cohort were T, A and T, respectively. There was a significantly higher proportion of the rs3077 and rs9277378 minor alleles (T and A, respectively) in the non-HBV infected controls than in the HBV carriers (p = 0.0040 and 0.0068, respectively). There was a trend of a higher proportion of the rs3128917 T allele in the non-HBV infected controls than in the HBV carriers (p = 0.054). The HBV clearance subjects had a significantly higher proportion of rs3077 T allele, rs9277378 A allele, and rs3128917 T allele than in the HBV carriers (p = 0.0083, 0.00011, and 0.00017 for rs3077, rs9277378 and rs3128917, respectively).

**Table 1:** Allelic difference and its association with chronicity and clearance of HBV infection.

SNP ID	Minor Allele	HBV carriers(2n = 1000)	Non-HBV infectedsubjects (2n = 490)	HBV Clearancesubjects (2n = 518)	OR (95% CI)*	p*	OR (95% CI)†	p†
rs3077	T	207 (20.7%)	141 (28.8%)	143 (27.6%)	0.67 (0.51–0.88)	0.0040	1.41 (1.09–1.82)	0.0083
rs9277378	A	242 (24.2%)	159 (32.5%)	176 (34.0%)	0.70 (0.54–0.91)	0.0068	1.61 (1.26–2.05)	0.00011
rs3128917	T	335 (33.5%)	202 (41.2%)	231 (44.6%)	0.78 (0.62–1.00)	0.054	1.54 (1.23–1.93)	0.00017

All logistic regression analyses were adjusted for age and sex.

* HBV carriers vs. non-HBV infected subjects.

† Clearance subjects vs. HBV carriers.

Genotype frequencies for the 3 SNPs were compared between the HBV carriers and non-HBV infected controls, as well as between the HBV carriers and HBV clearance group. The genotype distributions of the 3 study groups are listed in Table 2. Compared with the non-HBV infected controls, HBV carriers had a lower prevalence of the minor alleles of rs3077 and rs9277378, as shown by both the dominant-effect (homozygote minor+heterozygote vs. homozygote major) model (p = 0.0089 and 0.0162 for rs3077 and rs9277378, respectively) and the additive-effect (additive dosage of minor allele) model (*P* = 0.0036 and 0.0058 for rs3077 and rs9277378, respectively). There was also a lower frequency of the rs3128917 T allele in the HBV carriers when analyzed using the dominant-effect model (p = 0.0395), but the difference was only marginal when the additive-effect model was applied (p = 0.0561).

**Table 2:** Association of HLA-DP genotypes with chronicity and clearance of HBV infection.

SNP ID	Genotype/geneticmodel	HBV carriers (%) n = 500	Non-HBV infected subjects (%) n = 245	HBV Clearance subjects (%) n = 259	OR (95% CI)*	p*	OR (95% CI)†	p†
rs3077	CC	314 (62.8)	123 (50.2)	136 (52.5)	1.00	–	1.00	–
	TC	164 (33.0)	103 (42.0)	103 (39.8)	0.68 (0.48–0.97)	0.0312	1.31 (0.94–1.82)	0.109
	TT	21 (4.2)	19 (7.8)	20 (7.7)	0.41 (0.20–0.87)	0.0193	2.35 (1.20–4.58)	0.0125
	Dominant				0.64 (0.45–0.89)	0.0089	1.42 (1.04–1.95)	0.0284
	Additive				0.66 (0.50–0.87)	0.0036	1.42 (1.10–1.83)	0.0079
rs9277378	GG	283 (56.6)	109 (44.5)	115 (44.4)	1.00	–	1.00	–
	AG	192 (38.4)	113 (46.1)	112 (43.2)	0.69 (0.49–0.98)	0.0402	1.40 (1.00–1.94)	0.0475
	AA	25 (5.0)	23 (9.4)	32 (12.4)	0.43 (0.22–0.83)	0.0119	3.20 (1.79–5.71)	8.71×10^−5^
	Dominant				0.66 (0.47–0.93)	0.0162	1.61 (1.18–2.2)	0.0029
	Additive				0.68 (0.52–0.90)	0.0058	1.62 (1.27–2.07)	0.00011
rs3128917	GG	227 (45.4)	83 (33.9)	80 (30.9)	1.00	–	1.00	–
	TG	211 (42.2)	122 (49.8)	127 (49.0)	0.68 (0.47–0.98)	0.0395	1.64 (1.16–2.32)	0.0056
	TT	62 (12.4)	40 (16.3)	52 (20.1)	0.68 (0.41–1.14)	0.141	2.22 (1.40–3.52)	6.84×10^−4^
	Dominant				0.69 (0.49–0.98)	0.0395	1.79 (1.29–2.48)	0.00054
	Additive				0.79 (0.62–1.00)	0.0561	1.52 (1.22–1.90)	0.00024

All logistic regression analyses were adjusted for age and sex.

* HBV carriers vs. non-HBV infected subjects.

† HBV clearance subjects vs. HBV carriers.

Comparison was also made between the HBV carriers and HBV clearance subjects to test the association of these 3 SNPs with natural clearance of HBV infection. As shown in Table 2, rs3077 T allele, rs9277378 A allele, and rs3128917 T allele were associated with an increased chance of clearance of infection in both the dominant-effect model (rs3077: OR = 1.42, 95% confidence interval [CI] = 1.04–1.95, p = 0.0284; rs9277378: OR = 1.61, 95% CI = 1.18–2.2, p = 0.0029; and rs3218917: OR = 1.79, 95% CI = 1.29–2.48, p = 0.00054) and the additive-effect model (rs3077: OR: 1.42, 95% CI = 1.1–1.83, p = 0.0079; rs9277378: OR = 1.62 95% CI = 1.27–2.07, p = 0.00011; and rs3218917: OR = 1.52, 95% CI = 1.22–1.9, p = 0.00024).

Genotypic analysis showed that rs9277378 AA genotype might be most relevant to the clearance of HBV infection (OR = 3.20, p = 8.71×10^−5^; Table 2). Therefore we performed subgroup analysis to investigate the role of rs3077 and rs3128917 in the patients/subjects with rs9277378 GG genotype, which represent the genotype least likely to clear HBV infection. As shown in Table 2, 398 patients/subjects had rs9277378 GG genotype: 283 (56.6%) HBV carriers and 115 (44.4%) subjects with HBV clearance. Among these 398 patients/subjects, there was no significant difference between the HBV carriers and HBV clearance subjects in the proportion of the protective allele of rs3077 (A allele proportion = 9.2% vs. 11.3%, respectively; p = 0.727) and rs3128917 (T allele proportion = 13.8% vs. 17.4%, respectively; p = 0.254).

### Haplotype Analysis

LD information of these 3 SNPs for our 3 study groups is shown in Table S2. Haplotype analysis was also performed to assess the effect of the combination of these SNPs on HBV chronicity and clearance of HBV. Of the 8 possible haplotypes out of these 3 SNPs, 5 common haplotypes (with overall haplotype frequencies >0.05) were identified. As shown in Table 3, comparing to the haplotype containing all 3 risk alleles (CGG), the haplotypes TAT and CAT were associated with a higher chance of HBV clearance (for TAT: OR = 1.64, 95% CI = 1.21–2.24, p = 0.0013; and for CAT: OR = 1.98, 95% CI = 1.35–2.9, p = 0.00041). Since both haplotype CAT and TAT were associated with HBV clearance, we also performed haplotype analysis on only the last 2 SNPs (rs9277378 and rs3128917; both at *HLA-DPB1* gene). The haplotype AT was significantly associated with an increased chance of HBV clearance (OR = 1.70, 95% CI = 1.32–2.18, p = 3.66×10^−5^, with reference to haplotype GG).

**Table 3:** Haplotype association with chronicity and clearance of HBV infection, with the most common haplotype, CGG, as the reference.

Haplotype	HBV carriers (%)	Non-HBV infected subjects (%)	HBV Clearance subjects (%)	OR (95% CI)*	p*	OR (95% CI)†	p†
CGG	597 (59.7%)	256 (52.2%)	256 (49.6%)	1	–	1	–
TAT	147 (14.7%)	106 (21.6%)	108 (20.9%)	0.62 (0.45–0.86)	0.0044	1.64 (1.21–2.24)	0.0013
CAT	80 (8%)	49 (10%)	66 (12.8%)	0.70 (0.45–1.09)	0.116	1.98 (1.35–2.90)	0.00041
CGT	102 (10.2%)	41 (8.4%)	50 (9.7%)	1.15 (0.76–1.74)	0.495	1.07 (0.74–1.56)	0.458
TGG	53 (5.3%)	28 (5.7%)	30 (5.8%)	0.70 (0.42–1.17)	0.177	1.31 (0.80–2.13)	0.213

All logistic regression analyses were adjusted for age and sex.

SNP order of haplotype: rs3077, rs9277378, rs3128917.

* HBV carriers vs. non-HBV infected subjects.

† Clearance subjects vs. HBV carriers.

### Association between HLA-DP Polymorphisms and HBV Disease Activity

Among the 500 HBV carriers, 192 (38.4%) were asymptomatic inactive carriers (HBV DNA levels <2,000 IU/ml and persistently normal ALT for least 12 months). The active carriers were significantly older than the inactive carriers (mean age: 48.1±12.3 vs. 44.7±11.7 years, respectively; p = 0.002), and there was a higher percentage of male in the active carriers (66%) than in the inactive carriers (53%; p = 0.003). Association analysis showed that there were no significant differences in the allele frequency of rs3077, rs9277378, and rs3128917 between the active and inactive HBV carriers (p = 0.175, 0.240, and 0.656, respectively). Similarly, there were no significant genotypic differences between the active and inactive carriers when with the dominant model (p = 0.341, 0.411 and 0.495 for rs3077, rs9277378 and rs3128917, respectively) and additive model (p = 0.172, 0.229 and 0.663 for rs3077, rs9277378 and rs3128917, respectively) were applied. None of the haplotypes was associated with HBV disease activity (all p>0.05).

## Discussion

Recent GWAS studies have suggested that certain variations in the *HLA-DP* regions are associated with protection against chronic hepatitis B as well as viral clearance [13], [14], [15]. In the present study, we have studied 3 SNPs to extend our understanding of the association of these variations with HBV infection in Chinese population in Hong Kong and identified that the rs3077 T allele, rs9277378 A allele and rs3128917 T allele were protective for chronicity of HBV infection. While other studies have demonstrated that *HLA-DP* SNPs rs3077 and rs9277535 are strongly associated with chronic hepatitis B infection [13], [14], [15], [16], [17], [18], [19], [20], [21], to our knowledge, the present study is the first study to determine the association between rs9277378 and chronicity of HBV infection. Although it is possible that the authentic effect of rs9277378 polymorphism may be due to its high LD with rs9277535, our findings with rs9277378 suggested that more SNPs (or combination of SNPs) in the *HLA-DP* regions may be associated with HBV infection.

Data on the association of HLA-DP variations with chronic HBV infection are relatively scarce. In one study with 201 Caucasian chronic HBV carriers and 235 controls, the rs3077 T allele has also been identified to be protective against chronic HBV infection [18]. However, in that study, the rs3077 T protective allele was the major allele in the Caucasian cohort. This is consistent with the data from the HapMap project, which show that the frequencies of the protective alleles for rs3077 (T), rs9277378 (A) and rs3128917 (T) were higher in people with European ancestry than in the African and Asian populations [22]. Taken together, all these findings of *HLA-DP* genomic variations may shed light on the difference in the geographic distribution of HBV infection: it is possible that the lower prevalence of chronic HBV infection in the European/Caucasian populations is due to the higher prevalence of the protective *HLA-DP* alleles. Similarly, the high prevalence of chronic HBV infection in the Asian/African populations is likely due to the lower prevalence of the protective *HLA-DP* alleles. However, it should be noted that other factors, apart from HLA-DP variations, are also associated with chronicity of HBV infection. If the *HLA-DP* variations were the sole decisive factors for chronicity, the prevalence of chronic hepatitis B would have been much more than 10% in the Chinese. Moreover, a certain proportion of Asian/Chinese who possess the risk *HLA-DP* alleles may not have contacted HBV in their life time. Thus, many other factors, such as viral, environmental and other host genetic factors, are likely to be associated with chronicity of HBV infection. Nevertheless, the findings from the present as well as other genetic association studies, suggest that *HLA-DP* variations are probably one of the genetic factors which plays an important role in the development of chronicity of HBV infection.

Clearance of HBV infection is associated with a high level of CD4+ T cells response [24], [25]. HLA-DP molecules, belonging to HLA class II, are involved in antigen presentation to CD4+ T helper cells. The antigen-binding sites of HLA-DP molecules are highly polymorphic, and they play an important role in the physical binding of peptides and subsequent recognition by T-cell [26], [27]. While it can be expected that variations in the *HLA-DP* coding regions will affect antigen presentation and hence viral clearance, the 3 studied SNPs do not lie within the *HLA-DP* coding region. The SNP rs3077 lies in the 3′ untranslated region of *HLA-DPA1*, rs9277378 lies in the second intron of *HLA-DPB1*, and rs3128917 is located ∼2.5kb downstream of *HLA-DPB1* (Figure 1). As variations in these SNPs will not cause specific changes in the *HLA-DP* coding sequence, the effect of variations in these 3 SNPs on HLA-DP function and viral clearance is likely to be indirect. There are at least two possible mechanisms. Firstly, it is possible that variation in these SNPs may alter the expression of the *HLA-DP* genes, through the alternation of non-coding RNA sequence or microRNA binding site, as demonstrated in a recent study that variations in rs3077/rs3128917 and rs9277535 affect the expression of *HLA-DPA1* and *HLA-DPB1* respectively [28]. Secondly, as these SNPs are in a strong LD with the *HLA-DP* alleles, it is also likely that variations in these 3 SNPs reflect some yet to be identified variations in *HLA-DP* coding sequence [13], [16]. Thus variations in these 3 SNPs may be a marker for the variations in the *HLA-DP* coding sequence, which in turn affect antigen presentation of HBV-derived peptides and alter immune response and chronicity of infection. In fact, it has been demonstrated in a chimpanzee HBV infection model that the outcome of HBV infection is determined by the kinetics of viral spread and CD4 T-cell priming [29]. This suggests that the outcome of HBV infection can be influenced by the physical binding of HBV-derived peptides and their subsequent recognition by CD4 T-cell, which is dependent on *HLA-DP* polymorphism. The correlation between variations in *HLA-DPA1* and *HLA-DPB1* SNPs and the change in *HLA-DP* gene expression and molecule structure deserves a more thorough sequence analysis, and the functional roles of these polymorphisms remain to be studied.

Haplotype-based association analysis is more sensitive than individual SNP association analysis and can capture additional phenotype-related variants with a greater statistical power. This study found that both haplotypes TAT and CAT were associated with an increase chance of HBV clearance, with ORs of 1.64 and 1.98, respectively, both of which were greater than that of the individual SNPs (Table 1). However, there are two caveats. First, although the haplotype CAT showed the greatest OR of 1.98, its relatively greater 95% CI range and low overall haplotype frequency (0.097; data not shown) suggested that its effect on HBV clearance requires further investigations. Second, compared to the OR for individual alleles in the SNPs (for example, for rs9277378, OR = 1.61; Table 1), there was only a small increase in OR by the current haplotype analysis. In this current study, we found that the rs9277378 AA genotype might have the strongest association with HBV clearance (Table 2), and subgroup analysis indicated that the role of other protective SNPs in the rs9277378 GG subgroup was not significant. Therefore individual SNP analysis may be sufficient to provide information on the single most relevant and best-associated SNP with HBV clearance. Nevertheless, haplotype analysis may still have its value by increasing the statistical power in the association analysis and taking into account the effect of variants in other SNPs.

Given the greater genetic distance and weak LD between rs3077 (near *HLA-DPA1*) and the two other SNPs (rs9277378 and rs3128917; both near *HLA-DPB1*) and the relatively high LD between rs9277378 and rs3128917 (Table S2), it is possible that the two *HLA-DPB1* SNPs form one haplotype block while rs3077 belongs to a distinct haplotype block. Our finding that haplotype of the *HLA-DPB1* SNPs (rs9277378 and rs3128917) alone was associated with HBV clearance (OR = 1.70), independent on the effect of *HLA-DPA1* SNP rs3077, also pointed to this possibility. Although, in our present analysis, the effect of rs3077 alone on HBV clearance appeared to be less than that of the rs9277378-rs3128917 haplotype, it is likely that a more complex network or combination of more SNPs in the HLA region is associated with chronicity of HBV infection. Other recent studies have identified some SNPs in the *HLA-DQ* region which are also associated with susceptibility to HBV infection [14], [17]. The interaction between SNPs in the *HLA-DP* and *HLA-DQ* regions, their association with HBV infection in different populations, and their correlation with *HLA-DP* and *HLA-DQ* gene expression remain to be a challenging task to decode the genetic factors involved in HBV infection.

Another important finding from the present study is that we were not able to identify any association between *HLA-DP* genomic variations and HBV disease activity. This is consistent to other studies which also fail to identify any association between other SNPs in the *HLA-DP* region and HBV disease progression [18], [20]. Because only a limited number of SNPs was studied in our and other studies, more in-depth studies may be required to elucidate the association between *HLA-DP* variations and HBV disease activity. Similarly, the association between SNPs in the HLA regions and HCC development remains to be confirmed in different study cohorts. Two recent studies had identified 3 SNPs, rs2856718 (*HLA-DQA2/DQB1*), rs3077 (*HLA-DPA1*), and rs9272105 (*HLA-DQA1/DRB1*) to be associated with HBV-related HCC development [17], [30], while other studies failed to associate rs3077 and other HLA SNPs with HBV-related HCC development [15], [21], [31]. Detailed studies in different populations are needed to further elucidate the association between HLA genetic variations and HBV disease activity and HCC development.

In conclusion, we showed that *HLA-DP* SNP rs3077, rs9277378, and rs3128917 were individually associated with chronicity of HBV infection. Haplotype analysis revealed that haplotype TAT was strongly associated with HBV clearance. None of these 3 SNPs was associated with HBV disease activity.

## Supporting Information

Table S1 **Hardy-Weinberg calculations for all 3 polymorphisms in the HBV carriers, non-HBV infected and HBV clearance subject groups.**
(DOCX)

Table S2 **Linkage disequilibrium data in the HBV carriers, non-HBV infected and clearance subjects.**
(DOCX)
