# Supplementary material for: Role of HLA-DP Polymorphisms on Chronicity and Disease Activity of Hepatitis B Infection in Southern Chinese
Source: PLoS One. 2013 Jun 25;8(6):e66920. doi: 10.1371/journal.pone.0066920 (PMC3692552; doi:10.1371/journal.pone.0066920)
Supplement: Table S1 — Hardy-Weinberg calculations for all 3 polymorphisms in the HBV carriers, non-HBV infected and HBV clearance subject groups. (DOCX) [file pone.0066920.s001.docx]

**Table S1.** Hardy-Weinberg calculations for all 3 polymorphisms in the HBV carriers, non-HBV infected and HBV clearance subject groups

| SNP ID | Group | Chi-square | p |
| --- | --- | --- | --- |
| rs3077 | HBV carriers | 0.013 | 0.908 |
|  | Non-HBV infected subjects | 0.161 | 0.688 |
|  | HBV Clearance subjects | 0.007 | 0.935 |
|  | All | 0.00003 | 0.996 |
| rs9277378 | HBV carriers | 1.090 | 0.297 |
|  | Non-HBV infected subjects | 0.665 | 0.415 |
|  | HBV Clearance subjects | 0.338 | 0.561 |
|  | All | 0.200 | 0.655 |
| rs3128917 | HBV carriers | 1.397 | 0.237 |
|  | Non-HBV infected subjects | 0.186 | 0.666 |
|  | HBV Clearance subjects | 0.015 | 0.901 |
|  | All | 0.908 | 0.341 |

Genotypic distribution in Hardy-Weinberg equilibrium when p > 0.05.
