# Supplementary material for: Role of HLA-DP Polymorphisms on Chronicity and Disease Activity of Hepatitis B Infection in Southern Chinese
Source: PLoS One. 2013 Jun 25;8(6):e66920. doi: 10.1371/journal.pone.0066920 (PMC3692552; doi:10.1371/journal.pone.0066920)
Supplement: Table S2 — Linkage disequilibrium data in the HBV carriers, non-HBV infected and clearance subjects. (DOCX) [file pone.0066920.s002.docx]

**Table S2.** Linkage disequilibrium data in the HBV carriers, non-HBV infected and clearance subjects.

|  | HBV carriers | | | Non-HBV infected subjects | | | HBV Clearance subjects | | |
| --- | --- | --- | --- | --- | --- | --- | --- | --- | --- |
|  | rs3077 | rs9277378 | rs3128917 | rs3077 | rs9277378 | rs3128917 | rs3077 | rs9277378 | rs3128917 |
| rs3077 | -- | *0.581* | *0.517* | -- | *0.592* | *0.561* | -- | *0.562* | *0.481* |
| rs9277378 | 0.276 | -- | *0.895* | 0.295 | -- | *0.952* | 0.234 | -- | *0.990* |
| rs3128917 | 0.138 | 0.508 | -- | 0.182 | 0.621 | -- | 0.109 | 0.623 | -- |

For each study group, D′ data are in italic and on top right, and R^2^ data are shown in bottom left.
